# Impacts of complex electromagnetic radiation and low-frequency noise exposure conditions on the cognitive function of operators

**DOI:** 10.3389/fpubh.2023.1138118

**Published:** 2023-03-23

**Authors:** Peng Liang, Zenglei Li, Jiangjing Li, Jing Wei, Jing Li, Shenghao Zhang, Shenglong Xu, Zhaohui Liu, Jin Wang

**Affiliations:** ^1^Department of Rehabilitative Physioltherapy, The Second Affiliated Hospital of Air Force Medical University, Xi’an, China; ^2^Hospital of No. 95007 Unit of PLA, Guangzhou, China; ^3^Department of Anesthesiology, The Second Affiliated Hospital of Air Force Medical University, Xi’an, China; ^4^Ministry of Education Key Lab of Hazard Assessment and Control in Special Operational Environment, Department of Radiation Medical Protection, School of Military Preventive Medicine, Fourth Military Medical University, Xi’an, China; ^5^Department of Neurosurgery, The 940th Hospital of PLA Joint Logistics Support Force, Lanzhou, China; ^6^Department of Orthopaedics, The Second Affiliated Hospital of Air Force Medical University, Xi’an, China

**Keywords:** electromagnetic radiation, low-frequency noise, mental workload, fNIRS, n-back

## Abstract

**Background:**

Both electromagnetic radiation (EMR) and low-frequency noise (LFN) are widespread and influential environmental factors, and operators are inevitably exposed to both EMR and LFN within a complex exposure environment. The potential adverse effects of such exposure on human health must be considered seriously. This study aimed to investigate the effects of EMR and LFN on cognitive function as well as their interaction effect, which remain unclear.

**Methods:**

Sixty young male college students were randomly grouped and experiments were conducted with a 2 × 2 factorial design in a shielded chamber. Mental workload (MWL) levels of the study subjects were measured and assessed using the NASA-task load index (TLX) subjective scale, an n-back task paradigm, and the functional near-infrared spectroscopy (fNIRS) imaging technique.

**Results:**

For the 3-back task, the NASA-TLX subjective scale revealed a statistically significant main effect of LFN intensity, which enhanced the subjects’ MWL level (*F* = 8.716, *p* < 0.01). Behavioral performance revealed that EMR intensity (430.1357 MHz, 10.75 W/m^2^) and LFN intensity (0–200 Hz, 72.9 dB) had a synergistic interaction effect, and the correct response time was statistically significantly prolonged by the combined exposure (*F* = 4.343, *p* < 0.05). The fNIRS imaging technique revealed a synergistic interaction effect between operational EMR intensity and operational LFN intensity, with statistically significant effects on the activation levels in the left and right dorsolateral prefrontal cortex (DLPFC). The mean *β* values of DLPFC were significantly increased (L-DLPFC *F* = 5.391, *p* < 0.05, R-DLPFC *F* = 4.222, *p* < 0.05), and the relative concentrations of oxyhemoglobin in the DLPFC were also significantly increased (L-DLPFC *F* = 4.925, *p* < 0.05, R-DLPFC *F* = 9.715, *p* < 0.01).

**Conclusion:**

We found a statistically significant interaction effect between EMR (430.1357 MHz, 10.75 W/m^2^) and LFN (0–200 Hz, 72.9 dB) when simultaneously exposing subjects to both for 30 min. We conclude that exposure to this complex environment can cause a statistically significant increase in the MWL level of operators, and even alterations in their cognitive function.

## 1. Introduction

Electromagnetic radiation (EMR) is a form of radiation whereby electric and magnetic fields move in space as waves while effectively transferring energy and momentum (1). With the rapid development of communication technology, the rapidly increasing presence of an EMR environment has raised great concern about the adverse effects of EMR on human health (2, 3). Some studies have classified EMR from cell phones and other wireless devices as a possible human carcinogen (Class 2B) or even a probable human carcinogen (Class 2A) (4, 5). EMR exerts an effect on multiple systems of the whole body, and especially exerts effects on cognitive function (6). Additionally, low-frequency noise (LFN) refers to low-frequency broadband noise with a major component below 200 Hz and is a special environmental noise problem with widespread effects (7). An environmental health survey revealed that LFN caused 35% of all noise complaints among survey respondents (8). Among all noise components, LFN has the most pronounced effect on humans (9). For example, LFN can cause changes in cognitive function (10, 11). Since the public health problems caused by these two environmental factors put a great pressure on social and global economic development (3, 12), the World Health Organization (WHO) has listed water, air, noise, and EMR as the four major global pollution problems (13), and has highlighted the negative effects of LFN (14).

We note that a large body of survey evidence suggests that EMR and LFN often co-occur in both natural and operational conditions (15–18), and increasing attention is being paid to their adverse effects on operators’ cognitive function. However, few studies have been conducted on simultaneous exposure to these two environmental factors, and it is not clear whether they have an interaction effect.

With the rapid development of science and technology and the continuous change of occupational environments, the physical workload of operators has been greatly reduced, but the mental workload (MWL) is increasing. MWL is the result of a combination of factors, such as the level of effort a person exerts during an assignment and physiological and psychological demands during the assignment (19, 20), which is influenced by a combination of intrinsic mental stress and extrinsic environmental factors (21). Moreover, MWL is one of the most widely used concepts in human ergonomics research and practice (22, 23) and can be used to scientifically assess how well an operator performs current occupational operational tasks such as flying and driving (24, 25). Additionally, functional near-infrared spectroscopy (fNIRS) has been used for non-invasive monitoring of operators’ brain function during a variety of operational tasks, as it allows for more objective and sensitive measurement and assessment of the level of MWL than other methods, providing an important research method for brain and cognitive science (25–28).

This study investigated the effects of EMR and LFN on operators’ MWL using the fNIRS imaging technique and explored the main effects of EMR and LFN as well as their interaction effect on cognitive function. We hypothesized that EMR and LFN would exert averse main and synergistic effects on cognition. The findings provide an important theoretical and experimental basis for the formulation of environmental health standards for occupational exposures. However, in this experiment, the EMR and LFN intensities were only set at two levels, and the exposure time was only 30 min, so it cannot fully reveal the impact of these two environmental factors on brain cognitive function.

## 2. Materials and methods

### 2.1. Study subjects

The sample size of each group was estimated using G^*^POWER software (29). We planned to enroll at least 52 subjects, 13 per group, corresponding to a statistical power of 0.8068 and an alpha value of 0.05. Finally, 60 healthy young subjects were enrolled in this study, all of whom were male undergraduate or graduate students at the Air Force Medical University, 19–32 years of age (mean age 25.6 ± 4.4 years), right-handed, with normal hearing, and with no mental illness. Study subjects had not participated in a similar experiment within the last 6 months. The subjects were informed about the experiment and signed an informed consent form before the experiment was formally conducted. All subjects were randomly allocated into four groups using a random stratified sampling method, with 15 subjects in each group. All subjects passed a hearing test before enrollment (hearing threshold <25 dB HL at all standard frequencies from 0.25 to 8 kHz) and had no history of hearing-related disease.

This study was approved by the medical ethics committee of the First Affiliated Hospital of Air Force Military Medical University (approval number: KY20212098-F-1). This work was conducted in accordance with the principles of the Declaration of Helsinki and its later amendments.

### 2.2. Experimental design

A 2 × 2 factorial design was used for the experiment, where EMR was designed to have simulate pre-operational background radiation and operational radiation levels, and LFN was designed to have both pre-operational background noise and operational noise levels. The pre-operational background EMR intensity was 0.0002 W/m^2^, and the pre-operational background LFN intensity was 35.4 dB(A). Meanwhile, the operational EMR intensity was 430.1357 MHz with 10.75 W/m^2^, and the operational LFN was white noise of 0–200 Hz with 72.9 dB(A). Subjects were assigned to a pre-operational Control group, an LFN group, an EMR group, and a combined exposure group (Table 1), and the exposure duration for each group was 30 min.

**Table 1:** Schematic table of subject grouping in electromagnetic radiation (EMR) and low-frequency noise (LFN) environmental exposure experiment with a 2 × 2 factorial design.

LFN	EMR	
	0.0002 W/m^2^	10.75 W/m^2^
35.4 dB(A)	Control	EMR
72.9 dB(A)	LFN	Compound

The low-level exposure was pre-operational EMR exposure or pre-operational noise exposure in a laboratory setting, and the high-level exposure was operational EMR exposure or operational low-frequency white noise (LFWN) exposure. The Control group was exposed to pre-operational EMR and pre-operational noise in a laboratory setting. The LFN group was exposed to operational LFWN and pre-operational EMR. The EMR group was exposed to operational EMR and pre-operational LFWN. The Compound group was exposed to operational EMR and LFWN.

The frequency band 430–440 MHz is an important frequency band for radio communications in China and is often used in various types of operational conditions. In this study, the exposure intensity of operational EMR and LFN was set as the average of the intensity measured early on site by this study group under complex operational conditions, such as aircraft piloting, automobile piloting, and radar communication, in order to simulate the actual exposure environment. To ensure that each subject’s exposure intensity to EMR and LFN was as consistent as possible, we fixed the position and angle of the wooden chair where the subjects sat. The operational EMR was generated by the Chinese Xiaomi walkie-talkie (channel selection was 430.1357 MHz), which was fixed by a corresponding holder adjusted based on the individual variability of subjects. To avoid psychological cues from EMR to subjects, we chose the location of the walkie-talkie to be the back of the brain with the antenna midpoint constantly at a vertical distance of 20 cm from the center of the subjects’ hindbrain plane, and all subjects began the experiment with open walkie-talkie operation but only signal emission in the EMR and the combined exposure group. The 0–200 Hz low-frequency white noise (LFWN) was synthesized using Adobe Audition software, generated using the Finnish GENELEC 7050 CPM and 8,020 DPM. The sound output was fixed and distributed on both sides of the operant display at a distance of 100 cm from where the subject was located. All experiments were conducted daily from 10:00 to 12:00 and 15:00 to 18:00 in an EMR- and noise-shielded chamber. The exposure duration of each group was 30 min, and the temperature and humidity of the experimental environment were kept constant.

The monitoring of EMR intensity at the site of exposure included both the reference levels and the basic restrictions for exposure, the former with the purpose of assessing the level of exposure to EMR of the operating environment, and the latter with the purpose of assessing the magnitude of the effects of biological tissues subjected to EMR. Detection of reference levels for exposure was calibrated using the German NARDA NBM-550 EMR analyzer. Since our exposure site was relatively fixed, we chose the mean values during 6-min continuous examinations and determined the mean results after five repeated measurements. These were the reference levels for EMR exposure, which were 430.1357 MHz, 10.75 W/m^2^, and 62.20 V/m for the operational environment in this study. Specific energy absorption rate (SAR) testing was determined by the Switzerland SPEAG DASY5 professional system. With the aid of liquid substances and movable probes that simulate human tissue fluids, SAR values were calculated by the formula. In this study, the walkie-talkie was calibrated to give a head SAR of 3.34 Wkg^−1^ averaged over 10 g and a limbs SAR of 4.47 Wkg^−1^ averaged over 10 g, and the expanded uncertainty (95% confidence interval) was 20.18% for 10 g SAR (Supplementary Material). The exposure intensity was set lower than the reference levels and basic restrictions for occupational exposure at the corresponding frequencies in the ICNIRP guidelines (30).

The monitoring of LFN was calibrated using the Japanese RION NL-62 sound level meter and the LFN intensity was measured at the same exposure site as the EMR exposure. The average value obtained after three repeated measurements was the LFN intensity at the site of exposure. Considering the physical characteristics of sound, we chose the equivalent continuous sound pressure level for measurement and evaluation (31). The LFN intensity for the operational environment in the study was 72.9 dB (A), which was far below the occupational noise exposure limits specified by the Occupational Safety and Health Administration (OSHA) and the National Institute of Occupational Safety and Health (NIOSH) (12, 32).

### 2.3. Experimental procedures

The n-back task, first proposed by Kirchner, is commonly used to measure the level of MWL and is a common task paradigm in cognitive neuroscience research (33), wherein the level of MWL it elicits can be simultaneously reflected by fNIRS-based hemodynamic indicators (20, 34, 35). In this experiment, n-back tasks with four levels of difficulty were presented using the E-prime software to progressively elicit MWL. The n-back tasks were divided into four difficulty levels: 0-back, 1-back, 2-back, and 3-back, with the replicate blocks per level. Each block contained 26 randomly presented letters, where each subject had to decide whether the currently displayed letter was the same as the nth (*n* = 0, 1, 2, 3) preceding letter that had been displayed. Each letter was presented for 500 ms, and then each subject was given 1,500 ms to make a response.

If the target letter was found within this response time window, the subject had to press the “space” key as soon as possible. There was a 30-s interval between each two adjacent blocks, within which the subjects were asked to remain relaxed and focus on a “+” point on the screen in order to bring the brain activation level back to baseline levels. Each of the four tasks was performed from low to high levels. Guiding words were provided for task prompting before the task began. After the last block was completed, each subject was allowed to relax for 30 s, and then words indicating the end of the experiment were displayed. In addition, the formal experiment was preceded by block practice. During this practice, if the practice accuracy was so low that the subject was deemed as having failed to sufficiently focus on the task, the subject was asked to practice again until the accuracy was satisfactory, and then the formal experiment was conducted (34, 36). Next, eligible subjects were randomly grouped by a blinded method and then exposed to the two environmental factors in a manner designed for the group. Exposure was continuous throughout each n-back task and lasted approximately 30 min. The 0-back, 1-back, and 2-back tasks were carried out from low to high levels, thereby progressively eliciting MWL. Behavioral data (correct response time and correct response rate) were collected from the formal 3-back task, and the NASA-task load index (TLX) scale was completed immediately after the task. This scale consists of six items—mental demand, physical demand, time demand, self-performance, degree of effort, and degree of frustration—and can be used to standardize the assessment of MWL levels (37, 38). It has been widely used to assess MWL in a variety of operational conditions (39–41).

### 2.4. Functional near-infrared spectroscopy data acquisition and analysis

**Figure 1:**
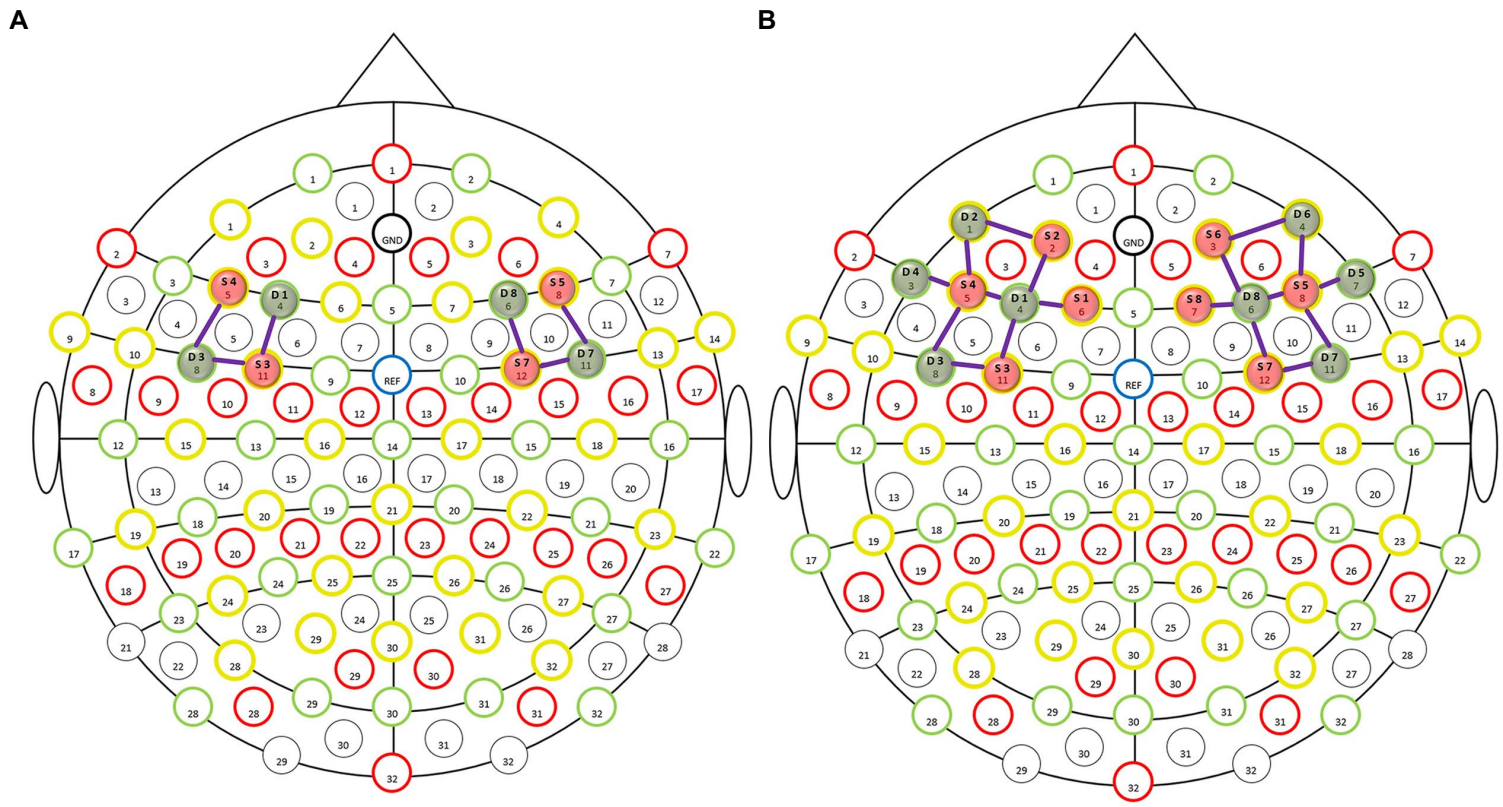
Layout of light sources and detectors in the dorsolateral prefrontal region of the enrolled subjects. The functional near-infrared spectroscopy (fNIRS) montage was visualized on the 10–20 EEG template, wherein the red solid balls present the light sources, green solid balls present the detectors, and the purple lines between the light sources and detectors present the optical detection channels. **(A)** fNIRS montage visualized on the 10–20 EEG template. Selected optodes and channels covering the dorsolateral prefrontal cortex (DLPFC). **(B)** Whole montage visualized on the 10–20 EEG template.

In this experiment, data acquisition was performed using a benchtop near-infrared (NIR) brain imaging system (NIRScout, NIRx, United States). The sites of the International 10–20 electroencephalography (EEG) system were covered using 128-port EasyCap positioning caps. With the line between Fpz and Iz on the skull aligned with the sagittal plane of the head, eight LED light sources and eight detectors were placed in the dorsolateral prefrontal region (Figure 1B) to collect signals from a total of 18 channels. To ensure the best sensitivity and signal-to-noise ratio, the spacing between the adjacent light source and detector was 3 cm and the sampling frequency was set to 7.8125 Hz (42). One study found that MWL elicited by the n-back task paradigm is reflected in the dorsolateral prefrontal cortex (DLPFC) (20, 43). Therefore, the regions of interest (ROIs) of the brain in this study were decided as follows: the left DLPFC (L-DLPFC, corresponding to the channels between F3-FC3, FC5-FC3, and FC5-F5 in the international 10–20 EEG system) and the right DLPFC (R-DLPFC, corresponding to the channels between F4-FC4, FC6-FC4 and FC6-F6 in the international 10–20 EEG system) (44, 45) (Figure 1A).

The acquired data were pre-processed using the NIRx software nirsLAB (v201904). According to the task paradigm of the experimental design, we first set the baseline level of interest, time point maker, and stimulation duration in the software, and then manually removed the data that had excessive noise fluctuations, followed by performing 0.01–0.20 Hz band-pass filtering on the remaining data to further remove noisy signals such as heartbeat, head movement, and slowly drifting signals (46).

Next, the coefficient of variation (CV) of the raw data’s signal-to-noise ratio was calculated in order to assess the reliability of the collected data (47, 48). Finally, the blood oxygen concentrations for each channel, including the relative levels of oxyhemoglobin (HBO), deoxyhemoglobin (HBR), and total hemoglobin, were converted and calculated according to the modified Beer–Lambert law (49). Modeling and convolutional operations were performed on data using a general linear model with hemodynamic functions and square wave functions, which led to an estimate of model parameter *β*, representing the degree of brain activation of subjects during the experimental task; the relative concentration of blood oxygen in a given ROI and the β value therein were taken as the means of all channel signals in the ROI (50, 51).

### 2.5. Statistical analysis

SPSS 26, GraphPad Prism 7, and Matlab 2013b statistical software were used for statistical analysis and plot generation. All experimental data, which were tested to verify a normal distribution and homoscedasticity, were expressed as means ± standard deviations. A 2 × 2 factorial design was used for the experiment. The main effect, interaction effect, and simple effect of EMR and LFN were calculated separately by two-factor analysis of variance (ANOVA), with *p* < 0.05 considered indicative of statistical significance.

## 3. Results

### 3.1. Subjective questionnaire results

**Figure 2:**
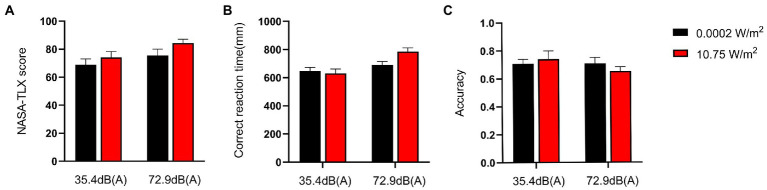
NASA-task load index (TLX) scores and behavioral performance of subjects completing the 3-back task under different exposure conditions. Scores are on a 0–100 scale. The score for each group was a weighted sum of the subscores that each respondent obtained for six items (mental demand, physical demand, time demand, self-performance, degree of effort, and degree of frustration), with a higher score indicating a higher level of mental workload (MWL). Correct response time was defined as the time interval from the instance when a subject saw the target letter to the instance when the subject pressed the space bar. Correct response rate was defined as the ratio of the number of times a subject pressed the space bar after seeing the target letter to the total number of times the subject pressed the space bar. Under a combined exposure condition, subjects had the highest scores and longest correct response time for completing the 3-back task, but the lowest correct response rate. **(A)** NASA-TLX scores when subjects completed the 3-back task under different exposure conditions. **(B)** Correct response time when subjects completed the 3-back task under different exposure conditions. **(C)** Correct response rate when subjects completed the 3-back task under different exposure conditions.

Subjects completed the NASA-TLX scale immediately after completing the 3-back task, with the total scores for each group shown in Figure 2A. The 2 × 2 factorial ANOVA revealed that the main effect of LFN was statistically significant, with *F* = 8.716, *p* < 0.01, and partial η^2^ = 0.135. The main effect of EMR was not statistically significant, with *F* = 0.684 and *p* > 0.05. The interaction effect between both exposures was likewise not statistically significant, with *F* = 1.801and *p* > 0.05 (Table 2).

**Table 2:** Statistical results of NASA-task load index (TLX) scores for subjects who completed the 3-back task under different exposure conditions (*N* = 60).

	0.0002 W/m^2^	10.75 W/m^2^	Main effect of LFN			Main effect of EMR			LFN*EMR		
	$\bar{\;x}\pm s$	$\bar{\;x}\pm s$	*F*	*P*	Partial η^2^	*F*	*P*	Partial η^2^	*F*	*P*	Partial η^2^
35.4 dB(A)	68.89 ± 16.13	66.78 ± 18.09	8.72	0.01^b^	0.14	0.68	0.41	0.01	1.8	0.19	0.031
72.9 dB(A)	75.49 ± 17.75	84.38 ± 10.28									

Higher scores indicate higher levels of mental workload (MWL). Factorial analysis of variance (ANOVA) was performed, with ^b^*p* < 0.01.

### 3.2. Behavioral performance results

Changes in the MWL level of subjects were elicited progressively through the n-back task paradigm, with the correct response time and correct response rate of subjects completing the 3-back task under different exposure conditions as shown in Figures 2B,C. A 2 × 2 factorial ANOVA revealed that, under a combined exposure condition, subjects’ correct response time increased statistically significantly upon completion of the 3-back task; the main effect of LFN intensity was statistically significant as well, with *F* = 13.421, *p* < 0.001, and a partial η^2^ = 0.193. The main effect of EMR intensity was not statistically significant, with *F* = 2.093 and *p* > 0.05; the interaction effect between the two factors was significant, with *F* = 4.343, *p* = 0.042, and partial η^2^ = 0.072.

Further analysis of the simple effect revealed that when exposing subjects to pre-operational background radiation, the simple effect of EMR intensity was not statistically significant, with *F* = 0.203, *p* > 0.05, and partial η^2^ = 0.004. However, when exposing subjects to operational LFN, the simple effect of EMR intensity was statistically significant, with *F* = 6.233, *p* = 0.016, and partial η^2^ = 0.100. When exposing subjects to low operational EMR, the simple effect of LFN intensity was not statistically significant, with *F* = 1.247, *p* = 0.269, and partial η^2^ = 0.022. When exposing subjects to high operational EMR, the simple effect of LFN intensity was statistically significant, with *F* = 16.517, *p* < 0.001, and partial η^2^ = 0.228 (Table 3). However, under a combined exposure condition, there was a decreasing trend in the correct response rate of subjects at the completion of the 3-back task, but the trend was not statistically significant. Under this exposure condition, the main effect of LFN intensity was not statistically significant, with *F* = 1.072 and *p* > 0.05. Moreover, the main effect of EMR intensity was not statistically significant, with *F* = 0.069 and *p* > 0.05. The interaction effect between the two factors was also not significant, with *F* = 1.079 and *p* > 0.05 (Table 4).

**Table 3:** Statistics for the correct response time for the 3-back task under different exposure conditions (*N* = 60).

	0.0002 W/m^2^	10.75 W/m^2^	Main effect of LFN			Main effect of EMR			LFN*EMR		
	$\bar{\;x}\pm s$ (ms)	$\bar{\;x}\pm s$ (ms)	*F*	*P*	Partial η^2^	*F*	*P*	Partial η^2^	*F*	*P*	Partial η^2^
35.4 dB(A)	647.50 ± 97.50	630.28 ± 120.03	13.42	0.001^b^	0.193	2.09	0.15	0.036	4.34	0.04^a^	0.072
72.9 dB(A)	690.15 ± 97.42	785.51 ± 101.80									

Correct response time was defined as the time interval from the instance when a subject saw the target letter to the instance when the subject pressed the space bar, with ^a^*p* < 0.05 and ^b^*p* < 0.01.

**Table 4:** Statistics for the correct response rate for the 3-back task under different exposure conditions (*N* = 60).

	0.0002 W/m^2^	10.75 W/m^2^	Main effect of LFN			Main effect of EMR			LFN*EMR		
	$\bar{\;x}\pm s$	$\bar{\;x}\pm s$	*F*	*P*	Partial η^2^	*F*	*P*	Partial η^2^	*F*	*P*	Partial η^2^
35.4 dB(A)	0.707 ± 0.125	0.740 ± 0.226	1.07	0.31	0.020	0.07	0.79	0.001	1.08	0.30	0.019
72.9 dB(A)	0.707 ± 0.170	0.651 ± 0.119									

Correct response rate was defined as the ratio of the number of times a subject pressed the space bar after seeing the target letter to the total number of times the subject pressed the space bar.

### 3.3. Functional near-infrared spectroscopy results

#### 3.3.1. Average *β* of ROIs

**Figure 3:**
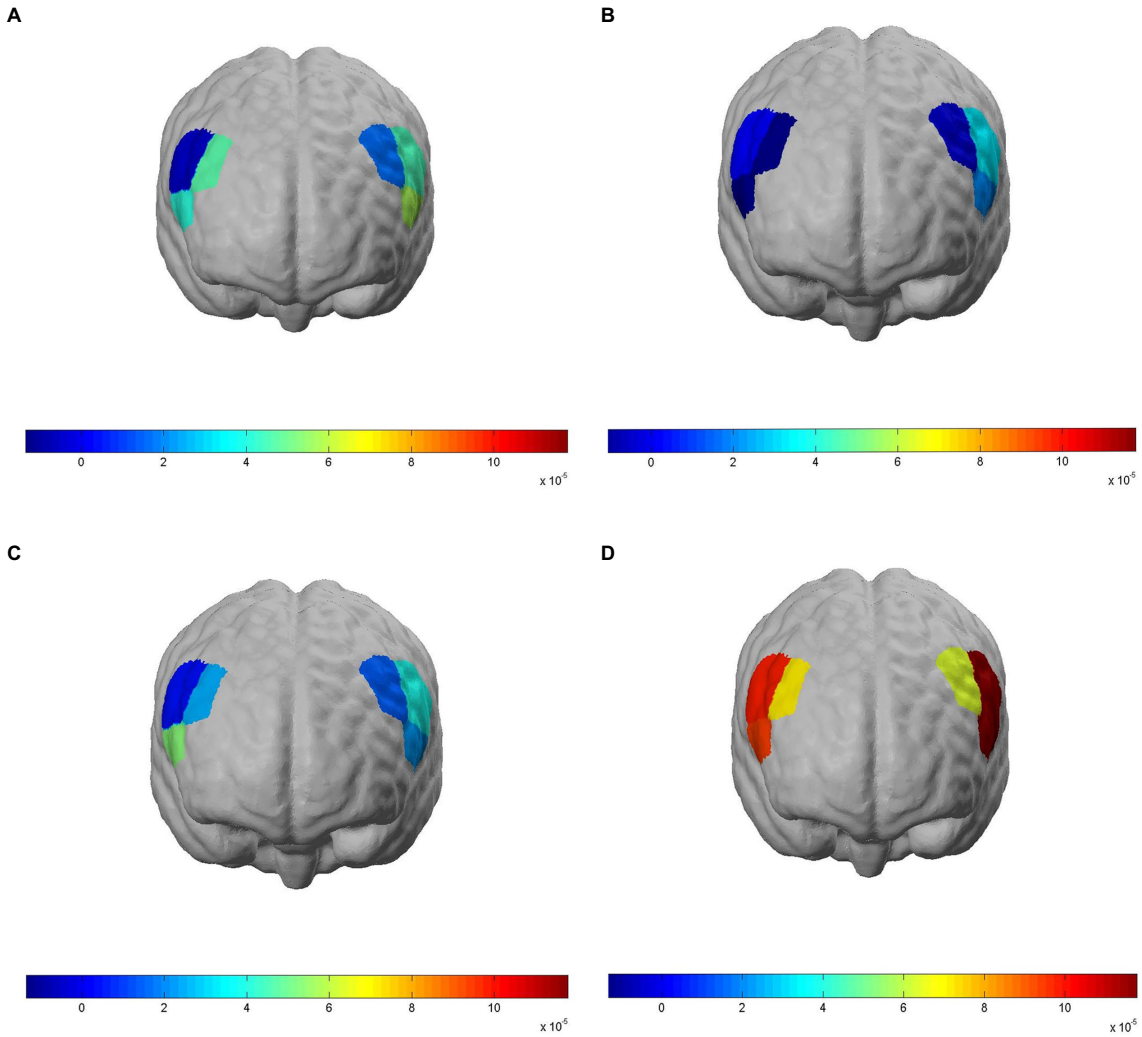
Brain activation in regions of interest (ROIs) when subjects completed the 3-back task under different exposure conditions. Brain ROIs are brain regions in the bilateral dorsolateral prefrontal cortex (DLPFC), and activation is represented by model-derived *β* values; red indicates a high degree of activation in brain regions and blue indicates a low degree of activation. **(A)** NASA-task load index (TLX) scores of the Control group when completing the 3-back task. **(B)** Correct response time of the low-frequency noise (LFN) group when completing the 3-back task. **(C)** Correct response rate of the electromagnetic radiation (EMR) group when completing the 3-back task. **(D)** Correct response rate of the Compound group when completing the 3-back task.

**Figure 4:**
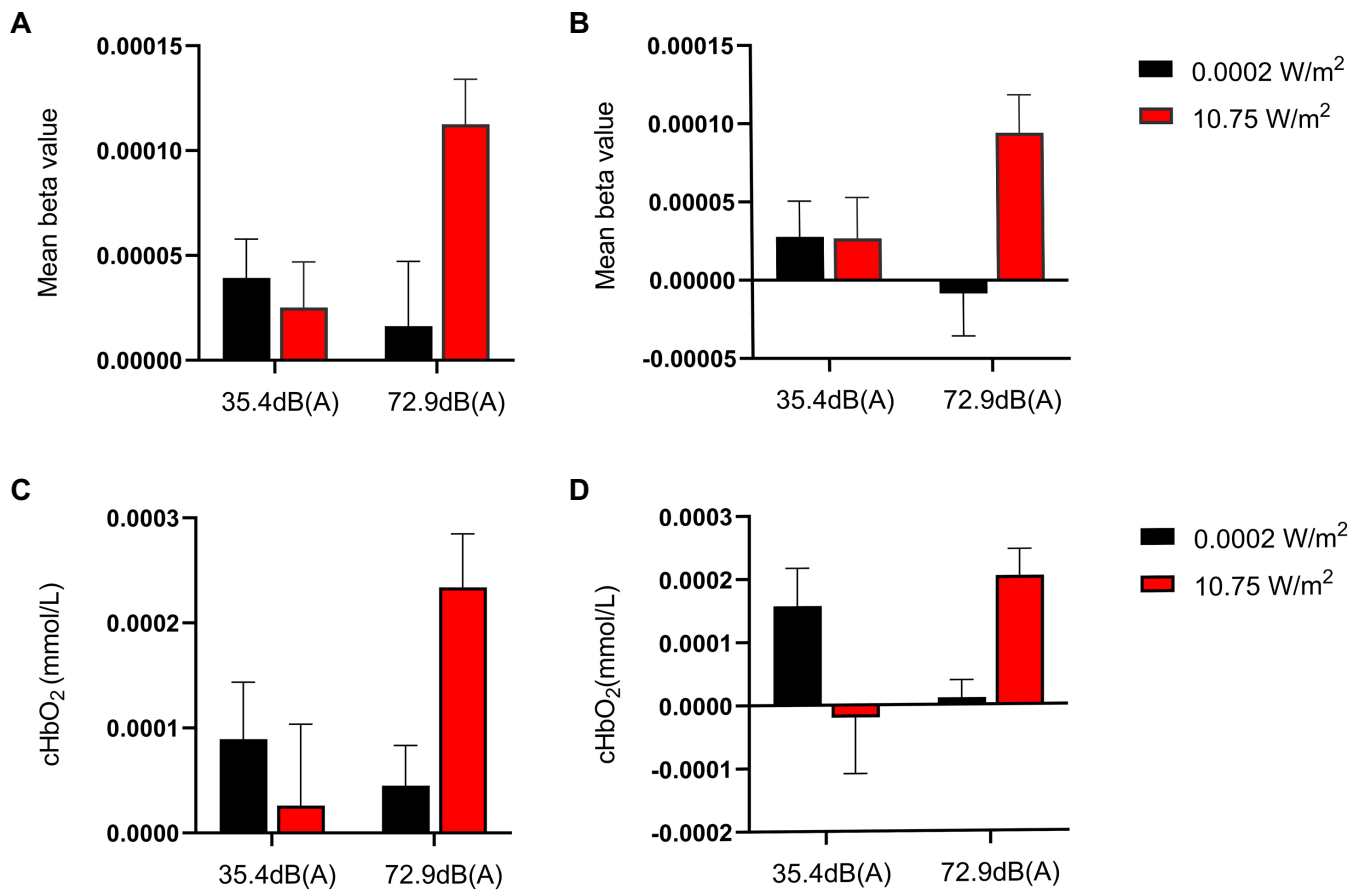
Mean *β* values and relative concentrations of oxyhemoglobin (HBO) in regions of interest (ROIs) when subjects completed the 3-back task under different exposure conditions. Larger *β* values indicate higher activation of brain regions. Among all exposure conditions, subjects completed the 3-back task with the highest activation in the ROI and the highest relative concentration of HBO under the combined exposure condition. **(A)** Mean *β* values for the left dorsolateral prefrontal cortex (L-DLPFC) in subjects who completed the 3-back task under different exposure conditions. **(B)** Mean *β* values for the right R-DLPFC in subjects who completed the 3-back task under different exposure conditions. **(C)** Relative HBO concentrations of L-DLPFC in subjects who completed the 3-back task under different exposure conditions. **(D)** Relative HBO concentrations of R-DLPFC in subjects who completed the 3-back task under different exposure conditions.

The *β* value derived from modeling and convolution operation using general linear models, hemodynamic functions, and square wave functions can represent the degree of activation of a particular brain region. Figure 3 shows the degree of brain activation in the right and left DLPFC when performing the 3-back task under different exposure conditions, with red indicating a high degree of activation in brain regions and blue indicating a low degree of activation. The 2 × 2 factorial ANOVA revealed that, under a combined exposure condition, subjects showed statistically significantly increased brain activation in the L-DLPFC upon completion of the 3-back task (Figure 4A), and that the interaction effect between LFN intensity and EMR intensity was statistically significant, with *F* = 5.391, *p* = 0.024, and partial η^2^ = 0.088. The main effect of LFN intensity was not statistically significant, with *F* = 1.855, *p* > 0.05, and partial η^2^ = 0.032. The main effect of EMR intensity was likewise not statistically significant, with *F* = 3.031, *p* > 0.05, and partial η^2^ = 0.051.

Further analysis of the simple effect revealed that, when exposing subjects to pre-operational background radiation, the simple effect of EMR intensity was not statistically significant, with *F* = 0.169, *p* > 0.05, and partial η^2^ = 0.003. However, the simple effect of EMR intensity was statistically significant when exposing subjects to operational LFN, with *F* = 8.254, *p* = 0.006, and partial η^2^ = 0.128. When exposing subjects to low operational EMR, the simple effect of LFN intensity was not statistically significant, with *F* = 0.461, *p* = 0.500, and partial η^2^ = 0.008. When exposing subjects to high operational EMR, the simple effect of LFN intensity was statistically significant, with *F* = 6.786, *p* = 0.012, and partial η^2^ = 0.108 (Table 5).

**Table 5:** Statistics showing the mean *β* values of the left dorsolateral prefrontal cortex (L-DLPFC) in subjects who completed the 3-back task under different exposure conditions (*N* = 60).

	0.0002 W/m^2^	10.75 W/m^2^	Main effect of LFN			Main effect of EMR			LFN*EMR		
	$\bar{\;x}\pm s$	$\bar{\;x}\pm s$	*F*	*P*	Partial η^2^	*F*	*P*	Partial η^2^	*F*	*P*	Partial η^2^
35.4 dB(A)	0.000039 ± 0.000072	0.000025 ± 0.000084	1.86	0.18	0.032	3.03	0.087	0.051	5.39	0.02^a^	0.088
72.9 dB(A)	0.000016 ± 0.000120	0.000113 ± 0.000084									

Higher *β* values indicate higher activation of brain regions, with ^a^*p* < 0.05.

Under a combined exposure condition, subjects showed increased brain activation in the R-DLPFC upon completion of the 3-back task (Figure 4B). Moreover, the interaction effect between LFN intensity and EMR intensity was statistically significant, with *F* = 4.222, *p* = 0.045, and partial η^2^ = 0.070. The main effect of LFN intensity was not statistically significant, with *F* = 0.380, *p* > 0.05, and partial η^2^ = 0.007. The main effect of EMR intensity was statistically significant, with *F* = 4.126, *p* = 0.047, and partial η^2^ = 0.069.

Further analysis of the simple effect revealed that, when exposing subjects to pre-operational background radiation, the simple effect of EMR intensity was not statistically significant, with *F* = 0.0003, *p* > 0.05, and partial η^2^ = 0.000005. However, when exposing subjects to operational LFN, the simple effect of EMR intensity was statistically significant, with *F* = 8.348, *p* = 0.005, and partial η^2^ = 0.130. When exposing subjects to low operational EMR, the simple effect of LFN intensity was not statistically significant, with *F* = 1.035, *p* = 0.313, and partial η^2^ = 0.018. When exposing subjects to high operational EMR, the simple effect of LFN intensity was not statistically significant, with *F* = 3.567, *p* = 0.064, and partial η^2^ = 0.060 (Table 6).

**Table 6:** Statistics for the mean *β* values of the right dorsolateral prefrontal cortex (R-DLPFC) in subjects who completed the 3-back task under different exposure conditions (*N* = 60).

	0.0002 W/m^2^	10.75 W/m^2^	Main effect of LFN			Main effect of EMR			LFN*EMR		
	$\bar{\;x}\pm s$	$\bar{\;x}\pm s$	*F*	*P*	Partial η^2^	*F*	*P*	Partial η^2^	*F*	*P*	Partial η^2^
35.4 dB(A)	0.000028 ± 0.000089	0.000027 ± 0.000101	0.38	0.54	0.007	4.13	0.047^a^	0.069	4.22	0.045^a^	0.07
72.9 dB(A)	−0.000009 ± 0.000105	0.000094 ± 0.000094									

Hsigher *β* values indicate higher activation of brain regions, with ^a^*p* < 0.05.

#### 3.3.2. Relative concentrations of cerebral oxyhemoglobin in ROIs

**Figure 5:**
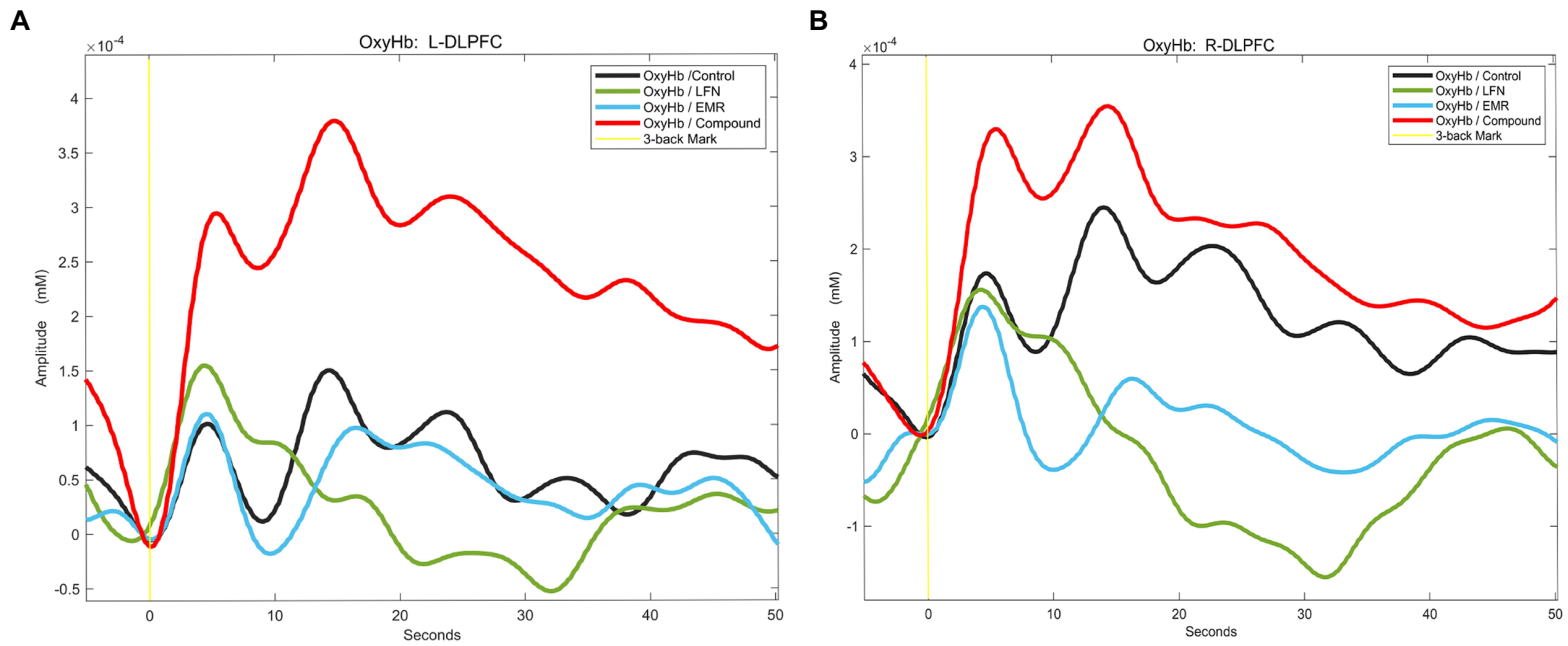
Relative concentration profiles of oxyhemoglobin (HBO) in the regions of interest (ROIs) in subjects performing the 3-back task under different exposure conditions. After the subjects started performing the 3-back task, HBO concentrations in the ROIs increased as the degree of MWL increased. Under a combined exposure condition, HBO concentrations increased most significantly. **(A)** Relative concentration profiles of HBO in the left dorsolateral prefrontal cortex (L-DLPFC) of subjects performing the 3-back task under different exposure conditions. **(B)** Relative concentration profiles of HBO in the right DLPFC (R-DLPFC) of subjects performing the 3-back task under different exposure conditions.

The relative concentrations of HBO were analyzed in the DLPFC region. It has previously been shown that changes in HBO have a better signal-to-noise ratio than changes in deoxyhemoglobin (HBR) in reflecting the level of neural activation in relevant brain regions and can better reflect the level of neural activation in the brain (52). Figure 5 shows the temporal profiles of relative HBO concentrations in the bilateral DLPFC during the 3-back task, demonstrating that under a combined exposure condition, the relative concentration profile of HBO in the bilateral DLPFC of the subjects was statistically significantly different from those under other exposure conditions.

The 2 × 2 factorial ANOVA revealed:

Under a combined exposure condition, subjects completed the 3-back task with a statistically significant increase in HBO concentrations in the L-DLPFC (Figure 4C). The interaction effect between LFN intensity and EMR intensity was statistically significant, with *F* = 4.925, *p* = 0.031, and partial η^2^ = 0.081. The main effect of LFN intensity was not statistically significant, with *F* = 1.198, *p* > 0.05, and partial η^2^ = 0.021. The main effect of EMR intensity was likewise not statistically significant, with *F* = 2.038, *p* > 0.05, and partial η^2^ = 0.035.

Further analysis of the simple effect revealed that, when exposing subjects to pre-operational background radiation, the simple effect of EMR intensity was not statistically significant, with *F* = 0.314, *p* > 0.05, and partial η^2^ = 0.006. When exposing subjects to operational LFN, the simple effect of EMR intensity was statistically significant, with *F* = 6.650, *p* = 0.013, and partial η^2^ = 0.106. When exposing subjects to low operational EMR, the simple effect of LFN intensity was not statistically significant, with *F* = 0.632, *p* = 0.430, and partial η^2^ = 0.011; however, when exposing subjects to high operational EMR, the simple effect of LFN intensity was statistically significant, with *F* = 5.492, *p* = 0.023, and partial η^2^ = 0.089 (Table 7).

**Table 7:** Statistics of relative oxyhemoglobin (HBO) concentrations in the left dorsolateral prefrontal cortex (L-DLPFC) of subjects who completed the 3-back task under different exposure conditions (*N* = 60).

	0.0002 W/m^2^	10.75 W/m^2^	Main effect of LFN			Main effect of EMR			LFN*EMR		
	$\bar{\;x}\pm s$ (mmol/L)	$\bar{\;x}\pm s$ (mmol/L)	*F*	*P*	Partial η^2^	*F*	*P*	Partial η^2^	*F*	*P*	Partial η^2^
35.4 dB(A)	0.000090 ± 0.000209	0.000045 ± 0.000148	1.20	0.28	0.021	2.04	0.16	0.035	4.93	0.03^a^	0.081
72.9 dB(A)	0.000026 ± 0.000301	0.000234 ± 0.000197									

Relative HBO concentration increased with increasing mental workload (MWL) level over a range, with ^a^*p* < 0.05.

Under a combined exposure condition, subjects showed a statistically significant increase in HBO concentration in the R-DLPFC upon completion of the 3-back task (Figure 4D). The interaction effect between LFN intensity and EMR intensity was statistically significant, with *F* = 9.715, *p* = 0.003, and a partial η^2^ = 0.148. The main effect of LFN intensity was not statically significant, with *F* = 0.016, *p* > 0.05, and partial η^2^ = 0.0003; the main effect of EMR intensity was not statistically significant, with *F* = 0.436, *p* > 0.05, and partial η^2^ = 0.008.

Further analysis of the simple effect revealed that when exposing subjects to pre-operational background radiation, the simple effect of EMR intensity was not statistically significant, with *F* = 3.018, *p* > 0.05, and partial η^2^ = 0.051. However, when exposing subjects to operational LFN, the simple effect of EMR intensity was statistically significant, with *F* = 7.134, *p* = 0.010, and partial η^2^ = 0.113. When exposing subjects to low operational EMR, the simple effect of LFN intensity was statistically significant, with *F* = 4.471, *p* = 0.039, and partial η^2^ = 0.074. When exposing subjects to high operational EMR, the simple effect of LFN intensity was also statistically significant, with *F* = 5.261, *p* = 0.026, and partial η^2^ = 0.086 (Table 8).

**Table 8:** Statistics of relative oxyhemoglobin (HBO) concentrations in the right dorsolateral prefrontal cortex (R-DLPFC) of subjects who completed the 3-back task under different exposure conditions.

	0.0002 W/m^2^	10.75 W/m^2^	Main effect of LFN			Main effect of EMR			LFN*EMR		
	$\bar{\;x}\pm s$ (mmol/L)	$\bar{\;x}\pm s$ (mmol/L)	*F*	*P*	Partial η^2^	*F*	*P*	Partial η^2^	*F*	*P*	Partial η^2^
35.4 dB(A)	0.000157 ± 0.000232	0.000012 ± 0.000108	0.02	0.90	0.0003	0.44	0.512	0.008	9.72	0.003^b^	0.148
72.9 dB(A)	−0.000020 ± 0.000346	0.000204 ± 0.000163									

Relative HBO concentrations increased with increasing mental workload (MWL) level over a range, with ^b^*p* < 0.01.

## 4. Discussion

Both EMR and LFN are prevalent and influential environmental factors; as operators are inevitably exposed to both EMR and LFN, this makes their work environment very complex. However, operators must perform important job duties under such complex environmental exposures, posing a great challenge for them. Few studies have investigated the biological effects of simultaneous exposure to these two environmental factors, and there are no clear reports regarding whether there is any interaction between them. Considering that exposure to EMR and LFN often occurs simultaneously, it is particularly important to understand whether each factor independently affects human health and whether their effects are additive or synergistic so as to effectively prevent their adverse effects on human health. We found a statistically significant interaction effect between EMR (430.1357 MHz, 10.75 W/m^2^) and LFN (0–200 Hz, 72.9 dB) when simultaneously exposing subjects to both for 30 min. We conclude that exposure to this complex environment, even where the exposure intensity is lower than the corresponding safety standards and limits, can cause a statistically significant increase in the operators’ MWL levels, and even alter their cognitive function. The adverse consequence of this interaction effect will be significant if the operators are exposed to it more intensely or for longer periods of time. Therefore, it is of great importance to avoid excessive exposure of operators to this complex environment and provide targeted protection. It is also urgent to conduct in-depth research to identify the effect pattern of EMR and LFN exposure on operators’ cognitive function as well as developing environmental health standards.

This experiment used a two factorial design (EMR levels: pre-operational background level, operational level) × 2 (LFN levels: pre-operational background level, operational level), in which the interaction effect between EMR and LFN was detected and validated using NASA-TLX subjective scores, 3-back task behavioral performance, and fNIRS-derived cerebral hemodynamics.

Research has shown that low-intensity LFN produces irritating subjective feelings and adversely affects mental performance, and that this adverse effect is more pronounced at high levels of MWL (53). High-intensity, prolonged LFN can meaningfully affect a person’s cognitive function and even lead to cognitive impairment (54). The NASA-TLX subjective scale showed that when individuals were exposed to high-intensity LFN (72.9 dB) for 30 min, they subjectively perceived that the noise environment significantly affected their work status and increased MWL; contrastingly, exposure to high-intensity EMR (10.75 W/m^2^) for 30 min did not have an obvious effect. This finding was consistent with the findings of other studies (53, 55, 56). This is likely either because the EMR exposure was not perceived by the subjects, or the intensity and duration of EMR exposure were so short that the exposure failed to have an adverse effect on the subjective evaluation of the exposure. Subjects’ behavioral performance on the 3-back task revealed that, when exposed to operational LFN, they needed longer response time and higher levels of MWL to achieve a similar correct response rate. Some studies with similar cognitive tasks have also found that noise exposure changes the correct response rate or time of subjects on a given task and causes higher levels of MWL (57–59). In this study, factorial ANOVA revealed that, during a 30-min period of simultaneous exposure to high-intensity LFN (72.9 dB) and high-intensity EMR (10.75 W/m^2^), (a) subjects’ total response time for the task increased significantly; (b) the correct response rate for the task tended to decrease, but not significantly; and (c) there was a significant synergistic interaction effect, and simultaneous exposure to LFN and EMR at certain intensity levels caused the highest MWL.

Currently, most studies have focused on the effects of EMR or LFN alone on human health and observed that single-factor exposure can lead to alterations in the central nervous system or brain cognitive function of operators. For example, one study observed that exposure to EMR affected human EEG alpha rhythms, which correspond to a purely cognitive signal and play an active role in attention, memory, and cognitive processes (60, 61). Another study using EEG techniques showed that an increase in task difficulty and noise exposure intensity led to a decrease in the correct response rate of the task and caused changes in EEG alpha, theta, and beta rhythms (58). With the progress of neuroimaging techniques, the research found that fNIRS technology enables better assessment of MWL levels in subjects under EMR conditions or ambient acoustic conditions by monitoring cerebral hemodynamics (62, 63). In our study, fNIRS data showed that, when simultaneously exposed to high-intensity LFN (72.9 dB) and EMR (10.75 W/m^2^) for 30 min, subjects’ L-DLPFC and R-DLPFC were activated upon completion of the 3-back task, and the relative concentrations of HBO in these two regions were increased. Our results indicated that combined exposure to high levels of these two environmental factors for 30 min significantly increased MWL levels in the subjects. This was consistent with the 3-back task behavioral performance, which provided objective evidence that there is a significant interaction effect between high operational LFN and EMR. Additionally, simultaneous exposure to both factors at high intensities can significantly increase MWL levels well above the MWL levels elicited by single-factor exposure.

This study had some limitations. First, only healthy young males were investigated in this study. The reason for focusing on this very specific test population was to avoid confounding effects of sex and age. However, it is unclear whether sex (males vs. females) and age (older vs. younger people) may affect susceptibility to EMR and LFN. This suggests a need for further studies with a larger sample size and more diverse population. Second, this study only observed the hemodynamics of the DLPFC of subjects under environmental exposure conditions, and the effects of EMR and LFN exposure on the hemodynamics of other brain regions need to be further explored. Finally, the EMR and LFN intensity were only set at two levels, and the exposure time was only 30 min, thereby making the intensity neither able to fully reflect the effect of exposure to the two environmental factors on brain cognitive function nor fully reveal the time-effect relationship and intensity-effect relationship.

All in all, we have presented a preliminary exploration which reveals the interaction effect between EMR and LFN, and there is abundant room for further progress on this topic, whether by expansion of the study population, diversification of exposure levels, or combination of EEG and fNIRS. Specifically, the application of emerging technologies at the level of assessing brain load requires further in-depth study.

## Data availability statement

The original contributions presented in the study are included in the article/Supplementary material, further inquiries can be directed to the corresponding authors.

## Ethics statement

The studies involving human participants were reviewed and approved by First Affiliated Hospital of Air Force Military Medical University (approval number: KY20212098-F-1). The patients/participants provided their written informed consent to participate in this study.

## Author contributions

PL wrote the manuscript. PL, ZLL, and JJL contributed to conception and design of the study. JWe organized the database. JL and SZ performed the statistical analysis. SX helped with statistical analysis. ZHL and Jwa directed the study.

## Funding

This work was supported by a Priority Project for Army Logistics Research (No. BSW17J029). The funder had no role in the design, conduct, or reporting of this work.

## Conflict of interest

The authors declare that the research was conducted in the absence of any commercial or financial relationships that could be construed as a potential conflict of interest.

## Publisher’s note

All claims expressed in this article are solely those of the authors and do not necessarily represent those of their affiliated organizations, or those of the publisher, the editors and the reviewers. Any product that may be evaluated in this article, or claim that may be made by its manufacturer, is not guaranteed or endorsed by the publisher.

## Abbreviations

ANOVA: analysis of variance
CV: coefficient of variation
DLPFC: dorsolateral prefrontal cortex
EEG: electroencephalography
EMR: electromagnetic radiation
fNIRS: functional near-infrared spectroscopy
HBO: oxyhemoglobin
HBR: deoxyhemoglobin
ICNIRP: International Commission on Non-Ionizing Radiation Protection
L-DLPFC: left dorsolateral prefrontal cortex
LFN: low-frequency noise
LFWN: low-frequency white noise
MWL: mental workload
NIOSH: National Institute of Occupational Safety and Health
NIR: near-infrared
OSHA: Occupational Safety and Health Administration
PET: positron emission tomography
R-DLPFC: right dorsolateral prefrontal cortex
ROI: region of interest
SAR: Specific energy absorption rate
WHO: World Health Organization
