# Supplementary material for: Impacts of complex electromagnetic radiation and low-frequency noise exposure conditions on the cognitive function of operators
Source: Front Public Health. 2023 Mar 23;11:1138118. doi: 10.3389/fpubh.2023.1138118 (PMC10076881; doi:10.3389/fpubh.2023.1138118)
Supplement: Supplementary file 1 [file Data_Sheet_1.DOCX]

Supplementary Material

Impacts of Complex Electromagnetic Radiation and Low-Frequency Noise Exposure Conditions on the Cognitive Function of Operators

**Peng Liang, Zenglei Li, Jiangjing Li, Jing Wei, Jing Li, Shenghao Zhang, Shenglong Xu, Zhaohui Liu* and Jin Wang***

***Correspondence:** Jin Wang: [wangjinn@fmmu.edu.cn](mailto:wangjinn@fmmu.edu.cn); Zhaohui Liu: [623364237@qq.com](mailto:623364237@qq.com)

# Supplementary Materials and Methods:

# 1.1 The SAR Measurement System

# This SAR Measurement System uses a computer-controlled 3-D stepper motor system (SPEAG DASY5 professional system). A E-field probe is used to determine the internal electric fields.

# The DASY5 system for performing compliance tests consists of the following items: A standard high precision 6-axis robot (Stabile RX family) with controller, teach pendant and software an arm extension for accommodation the data acquisition electronics (DAE).

# A dosimetric probe, i.e., an isotropic E-field probe optimized and calibrated for usage in tissue simulating liquid. The probe is equipped with an optical surface detector system.

# The DAE performs the signal amplification, signal multiplexing, AD-conversion, offset measurements, mechanical surface detection and collision detection, which is battery powered with standard or rechargeable batteries. The signal is optically transmitted to the electro-optical converter (EOC).

# The EOC performs the conversion between optical and electrical of the signals for the digital communication to DAE and for the analog signal from the optical surface detection. The EOC is connected to the measurement server.

# 1.2 Measurement procedure

# 1.2.1 Scanning procedure

# The “reference” and “drift” measurements were located at the beginning and end of the batch process, which measured the field drift at one single point in the liquid over the complete procedure. The SAR distribution at the exposed side of the head was measured at a distance of 4mm from the inner surface of the shell. The area covered the entire dimension of the head and the horizontal grid spacing was 15mm*15mm. Based on the area scan data, the area of the maximum absorption was determined by spline interpolation. Around this point, a volume of 30mm*30mm*30mm (fine resolution volume scan, zoom scan) was assessed by measuring 5x5x7 points. On this basis of this data set, the spatial peak SAR value was evaluated with the following procedure:

# The data at the surface was extrapolated, since the center of the dipoles is 2.0mm away from the tip of the probe and the distance between the surface and the lowest measuring point is 1.2mm. The extrapolation was based on a least square algorithm. A polynomial of the fourth order was calculated through the points in z-axes. This polynomial was then used to evaluate the points between the surface and the probe tip. The maximum interpolated value was searched with a straight-forward algorithm. Around this maximum the SAR values averaged over the spatial volumes (1g or 10g) were computed using the 3D-Spline interpolation algorithm. The volume was integrated with the trapezoidal algorithm. One thousand points were interpolated to calculate the average. All neighboring volumes were evaluated until no neighboring volume with a higher average value was found. The area and zoom scan resolutions specified must be applied to the SAR measurements. Probe boundary effect error compensation is required for measurements with the probe tip closer than half a probe tip diameter to the phantom surface. Both the probe tip diameter and sensor offset distance must satisfy measurement protocols; to ensure probe boundary effect errors are minimized and the higher fields closest to the phantom surface can be correctly measured and extrapolated to the phantom surface for computing 10-g SAR. Tolerances of the post-processing algorithms must be verified by the test laboratory for the scan resolutions used in the SAR measurements, according to the reference distribution functions specified in EN 62209-1/2.

# The power drift measurement job measures the field at the same location as the most recent power reference measurement job within the same procedure, and with the same settings. The indicated drift is mainly the variation of the DUT’s output power and should vary max ± 5 %.

# 1.2.2 Date storage

# The DASY software stored the acquired data from the data acquisition electronics as raw data (in microvolt readings from the probe sensors), together with all necessary software parameters for the data evaluation (probe calibration data, liquid parameters and device frequency and modulation data) in measurement files with the extension “.DAE”.

# 1.2.3 Date evaluation

# The SEMCAD software automatically executed the following procedures to calculate the field units from the microvolt readings at the probe connector. The parameters used in the evaluation were stored in the configuration modules of the software correctly. The first step of the evaluation was a linearization of the filtered input signal to account for the compression characteristics of the detector diode. The compensation depended on the input signal, the diode type and the DC-transmission factor from the diode to the evaluation electronics. From the compensated input signals the primary field data for each channel can be evaluated.

# 1.3 SAR System Verification

# 1.3.1 Tissue Simulate Liquid

# The head tissue dielectric parameters recommended by the IEEE SCC-34/SC-2 in P1528 are derived from planar layer models simulating the highest expected SAR for the dielectric properties and tissue thickness variations in a human head. Other head and body tissue parameters that have not been specified in P1528 are derived from the tissue dielectric parameters computed from the 4-Cole-Cole equations and extrapolated according to the head parameters specified in P1528. The dielectric parameters should be checked prior to assessment using the SPEAG DAK3.5 dielectric probe kit.

# The dielectric properties for the tissue simulate liquids were measured by using the SPEAG DAK3.5 dielectric probe kit in conjunction with Agilent E5071B Network Analyzer (300 KHz-8500 MHz). The conductivity (σ) and permittivity (ρ) are listed in Table 1. The temperature variation of the Tissue Simulate Liquids was 22±2°C.

# 1.3.2 SAR system check

# The daily system accuracy verification occurs within the flat section of the SAM phantom. A SAR measurement was performed to see if the measured SAR was within ± 10% from the target SAR values. The tests were conducted on the same days as the measurement of the equipment under test (EUT). The obtained results from the system accuracy verification are displayed in Table 2 (A power level of 250mW (below 3GHz) was input to the dipole antenna). During the tests, the ambient temperature of the laboratory was in the range 22±2°C, the relative humidity was in the range 60% and the liquid depth above the ear reference points was above 15±0.5 cm in all the cases. It is seen that the system is operating within its specification, as the results are within acceptable tolerance of the reference values.

# 1.4 Test results and measurement data

# For the PTT SAR tests, a communication link was set up with the test mode software for PTT mode test. The absolute radio frequency channel number (ARFCN) was allocated one channel in the case of 450 MHz during the test at the test frequency channel and the EUT was operated at the RF continuous emission mode. The channel should be tested at the 100% duty cycle. The obtained results from the system are displayed in Table 3.

# 1.5 Measurement uncertainty

# Measurements and results were all in compliance with the standards listed. All dates were recorded and maintained at the laboratory, performing the tests and measurement uncertainties were taken into account when comparing measurements to pass/fail criterial. The expanded uncertainty (95% confidence interval) was 20.18% for 10g SAR. The detailed measurements and results are displayed in Table 4.

# Supplementary Tables

**Supplementary Table 1.** **The conductivity (σ) and permittivity (ρ) of the tissue simulate liquids.** The dielectric properties for th**e** tissue simulate liquids were measured by using the SPEAG DAK3.5 dielectric probe kit in conjunction with Agilent E5071B Network Analyzer (300 KHz-8500 MHz).

| **Tissue**  **Type** | **Measured**  **Frequency** (**MHz**) | **Conductivity (σ)** | **Permittivity (ε_r_)** | **Conductivity Target (σ)** | **Permittivity Target (ε_r_)** | **Delta (σ)**  **(%)** | **Delta (ε_r_)**  **(%)** | **Limit**  **(%)** | **Liquid**  **Temp**  **(**℃**)** |
| --- | --- | --- | --- | --- | --- | --- | --- | --- | --- |
| 450 Head | 450 | 0.894 | 44.275 | 0.87 | 43.50 | 2.76 | 1.78 | ±5 | 22.1 |

**Supplementary Table 2. Summary system check results**. During the tests, the ambient temperature of the laboratory was in the range 22±2°C, the relative humidity was in the range 60% and the liquid depth above the ear reference points was above 15±0.5 cm in all the cases.

| **Validation Kit** | | **Measured SAR 1W** | **Measured**  **SAR 1W** | **Measured SAR** **(normalized to 1w)** | **Measured SAR** **(normalized to 1w)** | **Target SAR**  **(normalized**  **to 1w)**  **(±10%)** | **Target SAR**  **(normalized**  **to 1w)**  **(±10%)** | **Liquid**  **Temp**  **(**℃**)** |
| --- | --- | --- | --- | --- | --- | --- | --- | --- |
|  |  | 1g (W/kg) | 10g (W/kg) | 1g (W/kg) | 10g (W/kg) | 1-g(W/kg) | 10-g(W/kg) |  |
| D450V2 | Head | 1.11 | 0.729 | 4.44 | 2.916 | 4.53  (4.08-4.98) | 3.03  (2.73-3.33) | 22.1 |

**Supplementary Table 3. The main results from the SAR system.** The ARFCN was allocated one channel in the case of 450 MHz during the test at the test frequency channel and the EUT was operated at the RF continuous emission mode. The channel should be tested at the 100% duty cycle.

| **Test  mode** | **Ch. Space  (KHz)** | **Test Ch. /Freq.** | **SAR  (W/kg) 1-g** | **SAR  (W/kg)  10-g** | **Power Drift  (dB)** | **Conducted  Power  (dBm)** | **Tune   up  Limit  (dBm)** | **Scaled  factor** | **Scaled SAR  (W/kg)  10-g** | **Duty  cycle** | **Duty  cycle   50% scaled**  **SAR   (W/kg)**  **10-g** | **Liquid  Temp** | **SAR  limit (W/kg)  10-g** |
| --- | --- | --- | --- | --- | --- | --- | --- | --- | --- | --- | --- | --- | --- |
| **Head test data** | | | | | | | |  |  |  |  |  |  |
| FM | 12.5 | 10/434.4375 | 4.65 | 3.49 | -0.09 | 36.63 | 37.00 | 1.089 | 3.800 | 50% | 1.900 | 22.1 | 2.0 |
| FM | 12.5 | 1/430.1375 | 4.35 | 3.34 | 0.02 | 36.60 | 37.00 | 1.096 | 3.662 | 50% | 1.831 | 22.1 | 2.0 |
| FM | 12.5 | 20/439.4375 | 4.41 | 3.41 | 0.06 | 36.61 | 37.00 | 1.094 | 3.730 | 50% | 1.865 | 22.1 | 2.0 |
| **Limbs test data** | | | | | | | |  |  |  |  |  |  |
| FM | 12.5 | 10/434.4375 | 6.13 | 4.52 | 0.05 | 36.63 | 37.00 | 1.089 | 4.922 | 50% | 2.461 | 22.1 | 4.0 |
| FM | 12.5 | 1/430.1375 | 6.08 | 4.47 | 0.02 | 36.60 | 37.00 | 1.096 | 4.901 | 50% | 2.451 | 22.1 | 4.0 |
| FM | 12.5 | 20/439.4375 | 5.74 | 4.38 | 0.06 | 36.61 | 37.00 | 1.094 | 4.792 | 50% | 2.396 | 22.1 | 4.0 |

**Supplementary Table 4. Summary of the measurement uncertainty.** Measurements and results were all in compliance with the standards listed. All measurements and results were recorded and maintained at the laboratory and the expanded uncertainty (95% confidence interval) was 20.18% for 10g SAR.

| **a** | **b1** | **c** | **d** | **e=f (d, K)** | **f** | **g** | **i=C*g/e** | **i=C*g/e** | **k** |
| --- | --- | --- | --- | --- | --- | --- | --- | --- | --- |
| **Uncertainty Component** | **Section**  **in P1528** | **Tol**  **(%)** | **Prob. Dist** | **Div.** | **ci (1g)** | **ci (10g)** | **1-g**  **ui(%)** | **10-g**  **ui(%)** | **vi (Veff)** |
| **Measurement System** | | | | | | | | | |
| Probe Calibration(k=1) | E.2.1 | 6.3 | N | 1 | 1 | 1 | 6.30 | 6.30 | ∞ |
| Axial Isotropy | E.2.2 | 0.5 | R | √3 | 0.7 | 0.7 | 0.20 | 0.20 | ∞ |
| Hemispherical Isotropy | E.2.2 | 2.6 | R | √3 | 0.7 | 0.7 | 1.06 | 1.06 | ∞ |
| Boundary Effect | E.2.3 | 1.0 | R | √3 | 1 | 1 | 0.58 | 0.58 | ∞ |
| Linearity | E.2.4 | 0.6 | R | √3 | 1 | 1 | 0.35 | 0.35 | ∞ |
| System Detection Limits | E.2.4 | 0.25 | R | √3 | 1 | 1 | 0.14 | 0.14 | ∞ |
| Modulation Response | E.2.5 | 2.4 | R | √3 | 1 | 1 | 1.39 | 1.39 | ∞ |
| Readout Electronics | E.2.6 | 0.3 | N | 1 | 1 | 1 | 0.30 | 0.30 | ∞ |
| Response Time | E.2.7 | 0.0 | R | √3 | 1 | 1 | 0.00 | 0.00 | ∞ |
| Integration Time | E.2.8 | 2.6 | R | √3 | 1 | 1 | 1.50 | 1.50 | ∞ |
| RF Ambient Condition-Noise | E.6.1 | 3.0 | R | √3 | 1 | 1 | 1.73 | 1.73 | ∞ |
| RF Ambient Condition-Reflections | E.6.1 | 3.0 | R | √3 | 1 | 1 | 1.73 | 1.73 | ∞ |
| Probe Positioning-Mechanical Tolerance | E.6.2 | 1.5 | R | √3 | 1 | 1 | 0.87 | 0.87 | ∞ |
| Probe Positioning-with Respect to Phantom | E.6.3 | 2.9 | R | √3 | 1 | 1 | 1.67 | 1.67 | ∞ |
| Max. SAR Evaluation | E.5 | 1.0 | R | √3 | 1 | 1 | 0.58 | 0.58 | ∞ |
| **Test sample Related** | | | | | | | | | |
| Test sample Positioning | E.4.2 | 3.7 | N | 1 | 1 | 1 | 3.70 | 3.70 | 9 |
| Device Holder Uncertainty | E.4.1 | 3.6 | N | 1 | 1 | 1 | 3.60 | 3.60 | ∞ |
| Output Power Variation-SAR Drift Measurement | E.2.9 | 5 | R | √3 | 1 | 1 | 2.89 | 2.89 | ∞ |
| Output Power Variation-SAR Drift Measurement | E.6.5 | 0 | R | √3 | 1 | 1 | 0.00 | 0.00 | ∞ |
| **Phantom and Tissue Parameters** | | | | | | | | | |
| Phantom Uncertainty (Shape and Thickness Tolerances) | E.3.1 | 4 | R | √3 | 1 | 1 | 2.31 | 2.31 | ∞ |
| SAR Correction | E.3.2 | 1.9 | N | 1 | 1 | 0.84 | 1.90 | 1.60 | ∞ |
| Liquid Conductivity (Measurement Uncertainty) | E.3.3 | 0.23 | N | 1 | 0.78 | 0.71 | 0.18 | 0.163 | 5 |
| Liquid Permittivity (Measurement Uncertainty) | E.3.3 | -1.71 | N | 1 | 0.23 | 0.26 | -0.39 | -0.445 | 5 |
| Liquid Conductivity (Temperature Uncertainty) | E.3.4 | 4.2 | R | √3 | 0.78 | 0.71 | 1.89 | 1.72 | ∞ |
| Liquid Permittivity ((Temperature Uncertainty) | E.3.4 | 3.7 | R | √3 | 0.23 | 0.26 | 0.49 | 0.56 | ∞ |
| **Combined Standard**  **Uncertainty** |  |  |  | RSS |  |  | 10.17 | 10.09 | 430 |
| **Expanded Uncertainty**  **(95% Confidence Interval)** |  |  |  | *k*=2 |  |  | **20.34%** | **20.18%** |  |

References

1. EN50566: 2017: Product standard to demonstrate the compliance of wireless communication devices with the basic restrictions and exposure limit values related to human exposure to electromagnetic fields in the frequency range from 30 MHz to 6 GHz: hand- held and body mounted devices in close proximity to the human body

2. EN 62209-2: 2010: Human Exposure to Radio Frequency Fields from Handheld and Body-Mounted Wireless Communication Devices – Human models, Instrumentation, and Procedures - Part 2: Procedure to determine the specific absorption rate (SAR) for mobile wireless communication devices used in close proximity to the human body (frequency range of 30 MHz to 6 GHz)
